# Autophagy decreases alveolar macrophage apoptosis by attenuating endoplasmic reticulum stress and oxidative stress

**DOI:** 10.18632/oncotarget.13560

**Published:** 2016-11-24

**Authors:** Tao Fan, Lei Chen, Zhixin Huang, Zhangfan Mao, Wei Wang, Boyou Zhang, Yao Xu, Shize Pan, Hao Hu, Qing Geng

**Affiliations:** ^1^ Department of Thoracic Surgery, Renmin Hospital of Wuhan University, Wuhan, China; ^2^ Department of Gynecology and Obstetrics, Renmin Hospital of Wuhan University, Wuhan, China

**Keywords:** apoptosis, autophagy, endoplasmic reticulum stress, hypoxia-reoxygenation, ischaemia-reperfusion

## Abstract

To study the impact of autophagy on alveolar macrophage apoptosis and its mechanism in the early stages of hypoxia, we established a cell hypoxia-reoxygenation model and orthotopic left lung ischemia-reperfusion model. Rat alveolar macrophages stably expressing RFP-LC3 were treated with autophagy inhibitor (3-methyladenine, 3-MA) or autophagy promoter (rapamycin), followed by hypoxia-reoxygenation treatment 2 h, 4 h or 6 h later. Twenty Sprague-Dawley male rats were randomly divided into four different groups: no blocking of left lung hilum (model group), left lung hilum blocked for 1h with DMSO lavage (control group), left lung hilum blocked for 1 h with 100 ml/kg 3-MA (5 μmol/L) lavage (3-MA group), and left lung hilum blocked for 1 h with 100 ml/kg rapamycin (250 nmol/L) lavage (rapamycin group). Rapamycin decreased the unfolded protein response, which reduced endoplasmic reticulum stress-mediated apoptosis in the presence of oxygen deficiency. Rapamycin increased superoxide dismutase activities and decreased malondialdehyde levels, whereas 3-MA decreased superoxide dismutase activities and increased malondialdehyde levels. Thus, autophagy decreases alveolar macrophage apoptosis by attenuating endoplasmic reticulum stress and oxidative stress in the early stage of hypoxia *in vitro* and *in vivo*. This could represent a new approach to protecting against lung ischemia-reperfusion injury.

## INTRODUCTION

Autophagy is the process by which cytoplasmic material is delivered to lysosomes for degradation [1]. There are 3 types of autophagy: macroautophagy, microautophagy, and chaperone-mediated autophagy [2, 3]. Many stress pathways sequentially elicit autophagy and apoptosis within the same cell [4, 5]. A mass of compounds can induce autophagy for cell survival, or result in cell death. For example, glycyrrhetinic acid induces cytoprotective autophagy in non-small cell lung cancer (NSCLC) via the inositol-requiring enzyme 1α-c-Jun N-terminal kinase cascade. On the other hand, Clioquinol increased autophagic cell death in leukemia and myeloma cells by inhibiting the mTOR cascade [6, 7].

Highly conserved cysteine-aspartate proteases and caspases carry out transduction of apoptotic signaling pathways [15]. Caspase-3 is a downstream “effector” caspase, which is activated by initiator caspases, and has substrate specificity for the DEXD motif, a cleavage site similar to that found in many target proteins [16, 17]. Accumulation of caspase-3 in the nucleus indicates increased apoptotic activity [18].

The endoplasmic reticulum stress (ERS) that stimulates cellular apoptosis is reduced by hypoxia. The unfolded protein response (UPR) that follows ERS is anadaptive or protective response [19]. 78-kDa glucose-regulated/binding immunoglobulin protein (GRP78/BIP), X-box binding protein-1 (XBP1), and C/EBP–homologous protein (CHOP) are hallmarks for ERS [20–22]. CHOP and the caspase 12 in rodents (caspase 4 in humans) are recruited to promoten ERS-induced apoptosis [23, 24].

The oxidative stress theory of aging proposes that reactive oxygen species (ROS) generated by normal metabolism cause damage to macromolecules within the cell, which leads to cellular dysfunction and eventually organismal death [25–27]. Loss of superoxide dismutase (SOD) activity results in increased sensitivity to oxidative stress by lessening the ability of organism to detoxify ROS. Over expressing SOD reduced infarct size in acute myocardial infarction (AMI) by balancing ROS production and antioxidant defenses [28]. Malondialdehyde(MDA) is the end product of lipid peroxidation driven by ROS, which can contribute to the oxidative damage of proteins as it occurs under conditions of oxidative stress in ischemic heart disease and age-related diseases [29, 30].

The purpose of this study is to research the impact of autophagy on alveolar macrophage apoptosis in the early stage of hypoxia *in vitro* and *in vivo*, and to characterize its mechanism.

## RESULTS

### Effect of3-MA and rapamycin on RFP-LC3/NR8383cellviability

**Figure 1:**
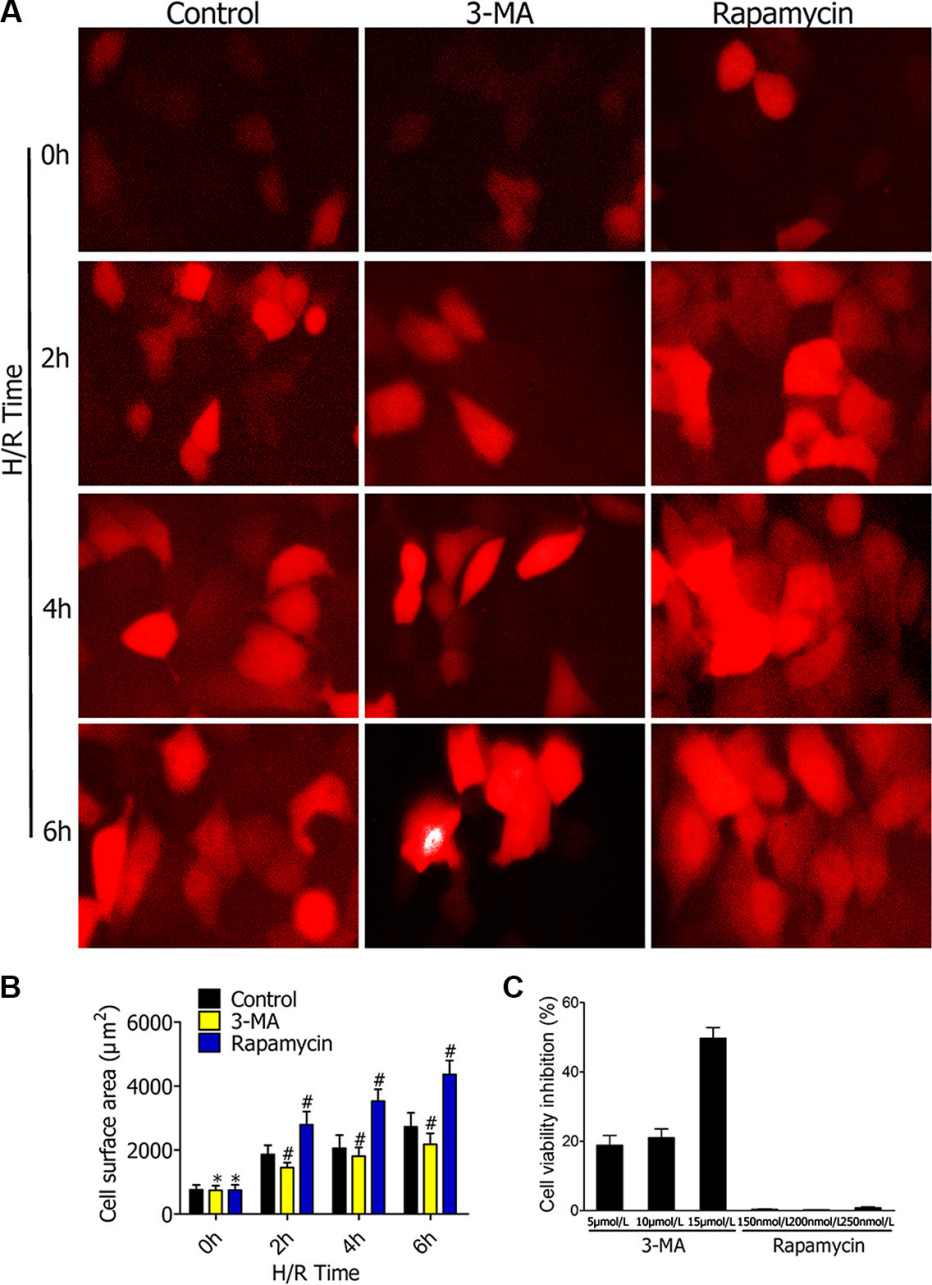
(**A**) Representative fluorescence microscopy images of RFP-LC3/NR8383 cells pretreated with 3-MA or rapamycin, followed by H/R treatment for 0 h, 2 h, 4 h, and 6 h. Bars represent 20 μm. (**B**) Quantitative results of the green cell surface area of RFP-LC3/NR8383 cells followed by H/R treatment for 0 h, 2 h, 4 h, and 6 h in response to DMSO, 3-MA, or rapamycin. (**C**) Impact of 3-MA and rapamycin on RFP-LC3/NR8383 cell viability. The cells are treated with different concentrations of 3-MA (5 μmol/L, 10 μmol/L, and 15 μmol/L) and rapamycin (150 nmol/L, 200 nmol/L, and 250 nmol/L) for 48 h. The control cells are treated with equal volume of DMSO. MTT assay is used to measure cell viability. The cell inhibition rate (%) is calculated by dividing by control values. (*p ≥ 0.05 compared to control at 0 h, ^#^*p* < 0.05 compared to control at 2 h, 4 h, and 6 h).

MTT assay was used to detect the effect of different concentrations of autophagy inhibitor3-MA and autophagy promoter rapamycin had on RFP-LC3/NR8383 cell viability. For 3-MA, the cell inhibition rate was 19.8%, 20.6%, and 49.2% at 5 μmol/L, 10 μmol/L, and 15 μmol/L, respectively (Figure 1C). For rapamycin, the cell inhibition rate was 0.4%, 0.1%, and 0.7% at 150 nmol/L, 200 nmol/L, and 250 nmol/L, respectively. Therefore, we chose 5 μmol/L 3-MA and 250 nmol/L rapamycin for further experiments.

### Fluorescence microscopy observation

TheRFP-LC3/NR8383cellswere observed under fluorescence microscopy to evaluate effects of 3-MA and rapamycinon autophagy formation following hypoxia-reoxygenation (H/R) treatment for 2 h, 4 h, and 6 h (Figure 1A). The red fluorescence indicated that RFP-LC3/NR8383cells were successfully constructed. The cellular surface areas were measured by immunostaining after pretreating with DMSO, 3-MA (5 μmol/L), and rapamycin (250 nmol/L), followed by H/R treatment for 0 h, 2 h, 4 h, and 6 h (Figure 1B). The measured surface areas of the red RFP-LC3/NR8383 cells indicated that 3-MA inhibited the expression of autophagy marker protein LC3. On the contrary, rapamycin promoted the expression of autophagy marker protein LC3.

### Autophagy activity was inhibited by 3-MA and strengthened by rapamycin

**Figure 2:**
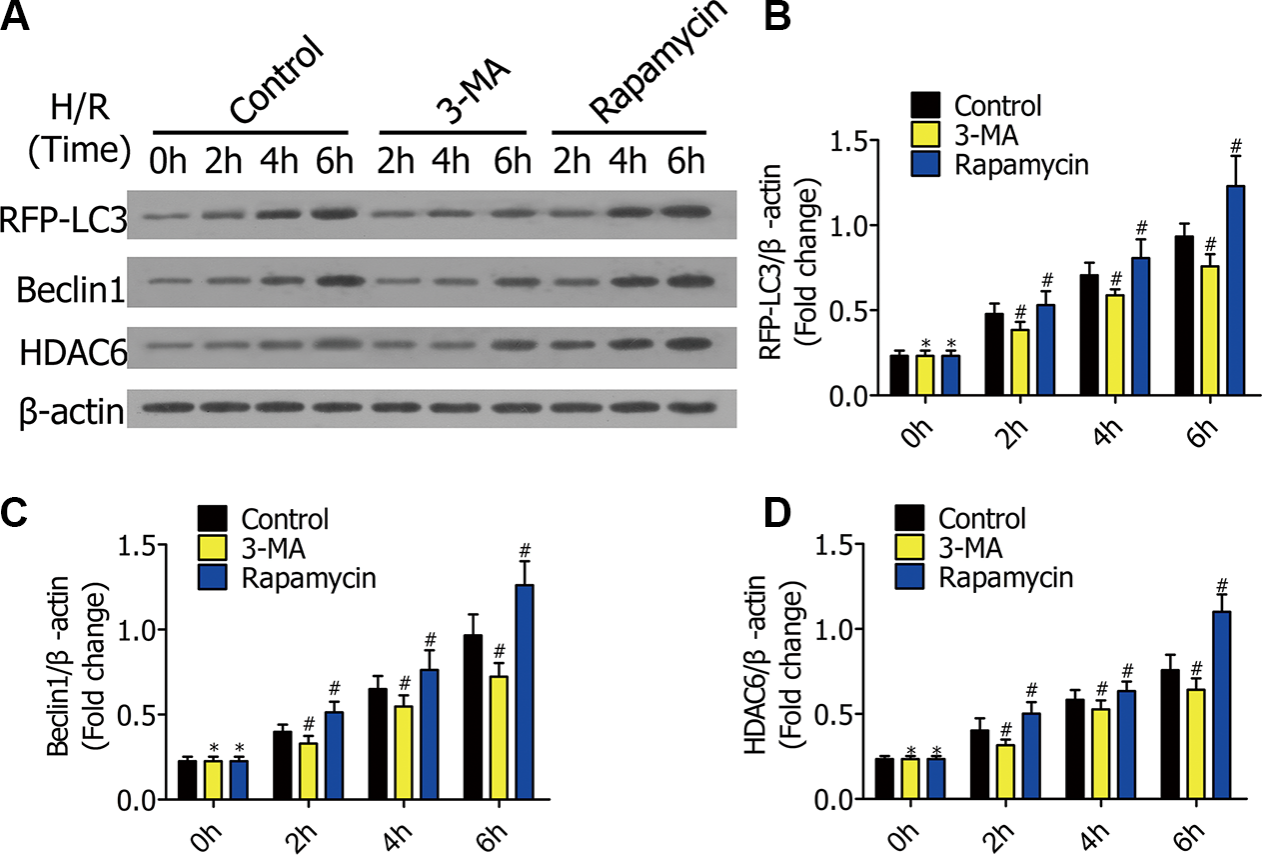
(**A**) Western blots showing the protein of RFP-LC3, Beclin1, and HDAC6 in RFP-LC3/NR8383 cells pretreated with DMSO, 3-MA, and rapamycin, followed by H/R treatment for 0 h, 2 h, 4 h, and 6 h. (**B**–**D**) The protein of RFP-LC3, Beclin1, and HDAC6 in RFP-LC3/NR8383 cells were quantified and analyzed in the indicated groups. (*p ≥ 0.05 compared to control at 0 h, ^#^*p* < 0.05 compared to control at 2 h, 4 h, and 6 h).

To verify3-MA and rapamycin can regulate autophagy, we measured the expression of autophagy-related genes in RFP-LC3/NR8383 cells. The cells were pretreated with DMSO, 3-MA (5 μmol/L), and rapamycin (250 nmol/L), followed by H/R treatment for 0 h, 2 h, 4 h, and 6 h. Western blotting results showed the protein levels of RFP-LC3, Beclin1, and HDAC6 in RFP-LC3/NR8383 cells (Figure 2A). The protein levels of RFP-LC3, Beclin1, and HDAC6were quantified and analyzed in the indicated groups (Figure 2B, 2C, 2D). The protein levels of RFP-LC3, Beclin1, and HDAC6 in the 3-MA group were lower than the control group, while they were higher in the rapamycin group.

### Caspase-3 expression is increased by 3-MA and decreased by rapamycin

**Figure 3:**
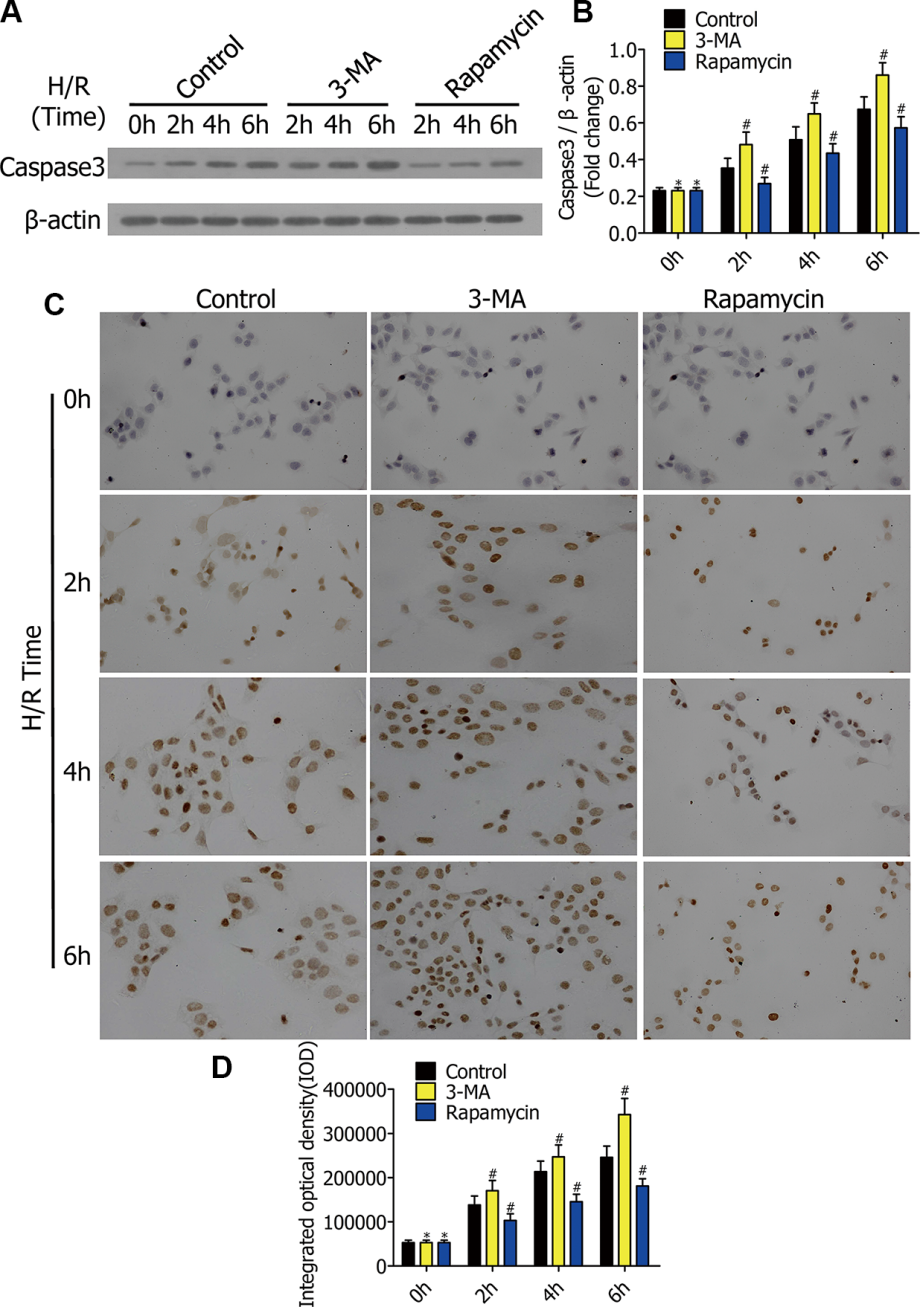
(**A**) Western blots showing caspase-3 protein in RFP-LC3/NR8383 cells pretreated with DMSO, 3-MA, and rapamycin, followed by H/R treatment for 0 h, 2 h, 4 h, and 6 h. (**B**) Caspase-3 protein was quantified and analyzed in the indicated groups. (**C**) Representative immunohistochemical staining images in RFP-LC3/NR8383 cells pretreated with DMSO, 3-MA, and rapamycin, followed by H/R treatment for 0 h, 2 h, 4 h, and 6 h. (**D**) Immunohistochemistry analysis of the protein expressions of caspase-3 in the indicated groups. (*p ≥ 0.05 compared to control at 0 h, ^#^*p* < 0.05 compared to control at 2 h, 4 h, and 6 h).

To research the impact of autophagy on alveolar macrophage apoptosis in the early stage of H/R, we preliminarily detected the caspase-3 level *in vitro*. RFP-LC3/NR8383 cells were pretreated with DMSO, 3-MA (5 μmol/L), and rapamycin (250 nmol/L), followed by H/R treatment for 0 h, 2 h, 4 h, and 6 h. Western blotting results showed the protein levels of caspase-3 in RFP-LC3/NR8383 cells (Figure 3A). The protein levels of caspase-3 were quantified and analyzed in the indicated groups (Figure 3B). Following H/R treatment for 0 h, 2 h, 4 h, and 6 h, the 3-MA pretreated cells displayed increased caspase-3 levels, while the rapamycin pretreated cells showed decreased levels. Immunohistochemical staining of RFP-LC3/NR8383 cells was used for further evaluating the expression of caspase-3 (Figure 3C). Immunohistochemistry results were similar to western blotting results, which indicated that 3-MA enhanced caspase-3 expression, while rapamycin reduced caspase-3 expression in early stages of H/R in RFP-LC3/NR8383 cells (Figure 3D).

### Enhanced autophagy decreased cell apoptosis and cell viability inhibition

**Figure 4:**
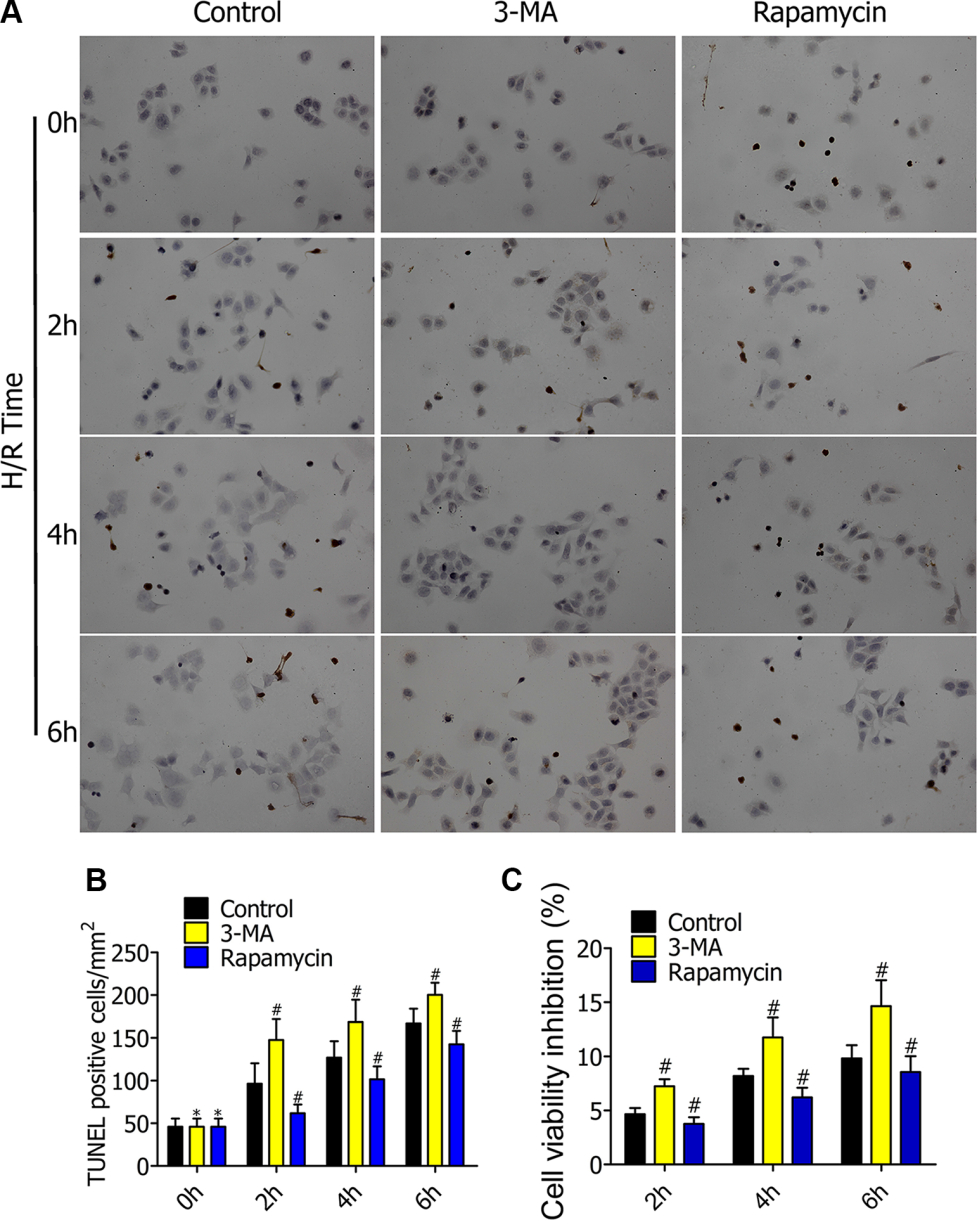
(**A**) TUNEL staining of RFP-LC3/NR8383 cells showed the apoptotic death of nuclear cells of alveolar macrophages in control, 3-MA, and rapamycin groups. (**B**) Percentage of TUNEL (+) cells (apoptosis cells) in TUNEL staining. (**C**) MTT assaying of alveolar macrophage viability inhibition ratio in control, 3-MA, and rapamycin group.(*p ≥ 0.05 compared to control at 0 h, #p < 0.05 compared to control at 2 h, 4 h, and 6 h)

To further determine the impact of autophagy on alveolar macrophage apoptosis and cell viability in early stages of H/R, we pretreated RFP-LC3/NR8383 cells with DMSO, 3-MA (5 μmol/L), and rapamycin (250 nmol/L), followed by H/R treatment for 0h, 2h, 4h, and 6h. TUNEL staining of RFP-LC3/NR8383 cells showed the apoptotic death of nuclear cells of alveolar macrophages in the control, 3-MA, and rapamycin groups (Figure 4A). The number of apoptotic cells was estimated by TUNEL. Compared to the control group, the percentage of TUNEL (+) cells (apoptosis cells) was higher in the 3-MA group, and lower in the rapamycin group (Figure 4B). MTT assay was used to detect the effect of different degrees of autophagy on RFP-LC3/NR8383 cells viability. Compared to the control group, the cell viability inhibition ratio was higher in the 3-MA group, and lower in the rapamycin group (Figure 4C).

### Enhanced autophagy decreased ERS in early stages of H/R

**Figure 5:**
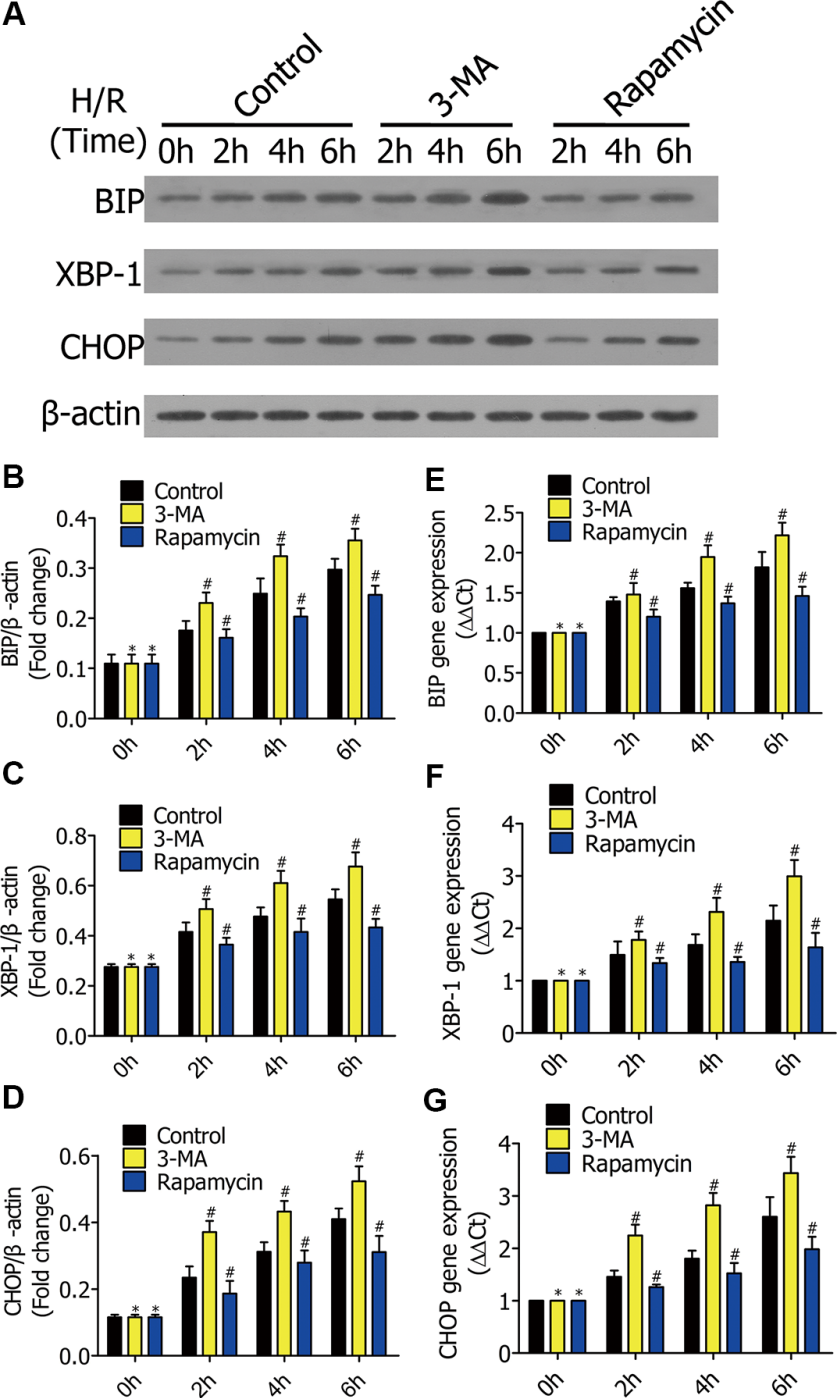
(**A**) Western blots showing the protein of BIP, XBP-1, and CHOP in RFP-LC3/NR8383 cells pretreated with DMSO, 3-MA, and rapamycin, followed by H/R treatment for 0 h, 2 h, 4 h, and 6 h. (**B**–**D**) The protein of BIP, XBP-1, and CHOP in RFP-LC3/NR8383 cells were quantified and analyzed in the indicated groups. (**E**–**G**) Real-time PCR showing the expression of BIP, XBP-1, and CHOP in the indicated groups. Results presented as (2–ΔΔCt) ± SD. (*p ≥ 0.05 compared to control at 0 h, ^#^*p* < 0.05 compared to control at 2 h, 4 h and 6 h).

To research the mechanism of how enhanced autophagy decreases cell apoptosis in early stages of H/R, we measured ERS-related expression. RFP-LC3/NR8383 cells were pretreated with DMSO, 3-MA (5 μmol/L), and rapamycin (250 nmol/L), followed by H/R treatment for 0 h, 2 h, 4 h, and 6 h. Western blotting results showed the protein levels of BIP, XBP-1, and CHOP (Figure 5A), which were quantified and analyzed in the indicated groups (Figure 5B, 5C, 5D). Real-time PCR further showed expression of the three genes (5E, 5F, 5G). The levels of BIP, XBP-1, and CHOP levels were higher in the 3-MA group than the control group, but lower in the rapamycin group.

### Effect of autophagy on superoxide dismutase activity and malondialdehyde

**Figure 6:**
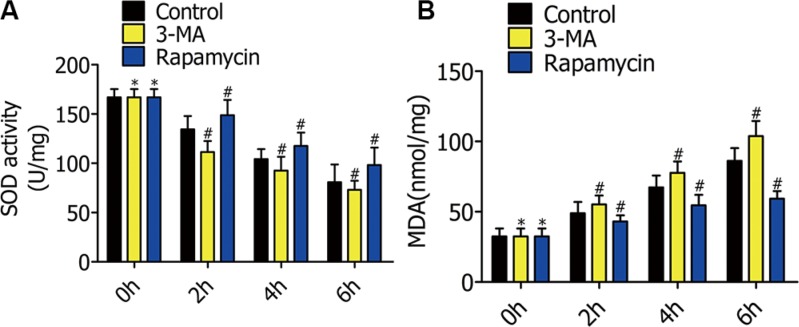
(**A**) Superoxide dismutase (SOD) activity in RFP-LC3/NR8383 cells pretreated with DMSO, 3-MA, and rapamycin, followed by H/R treatment for 0 h, 2 h, 4 h, and 6 h. (**B**) Malondialdehyde (MDA) content in RFP-LC3/NR8383 cells pretreated with DMSO, 3-MA, and rapamycin, followed by H/R treatment for 0 h, 2 h, 4 h, and 6 h. (*p ≥ 0.05 compared to control at 0 h, ^#^*p* < 0.05 compared to control at 2 h, 4 h, and 6 h).

To explore the mechanisms of autophagy reducing alveolar macrophage apoptosis in oxygen deficiency, we measured the MDA content and SOD activity to estimate oxidative stress level. RFP-LC3/NR8383 cells pretreated with DMSO, 3-MA (5 μmol/L), and rapamycin (250 nmol/L) were subjected to H/R treatment for 0 h, 2 h, 4 h, and 6 h. SOD activity in the 3-MA group was lower than the control group, but higher in the rapamycin group (Figure 6A). Converse to the SOD levels, MDA concentrations decreased with increased autophagy (Figure 6B).

### In I/R, caspase-3 expression increased by 3-MA, decreased by rapamycin

**Figure 7:**
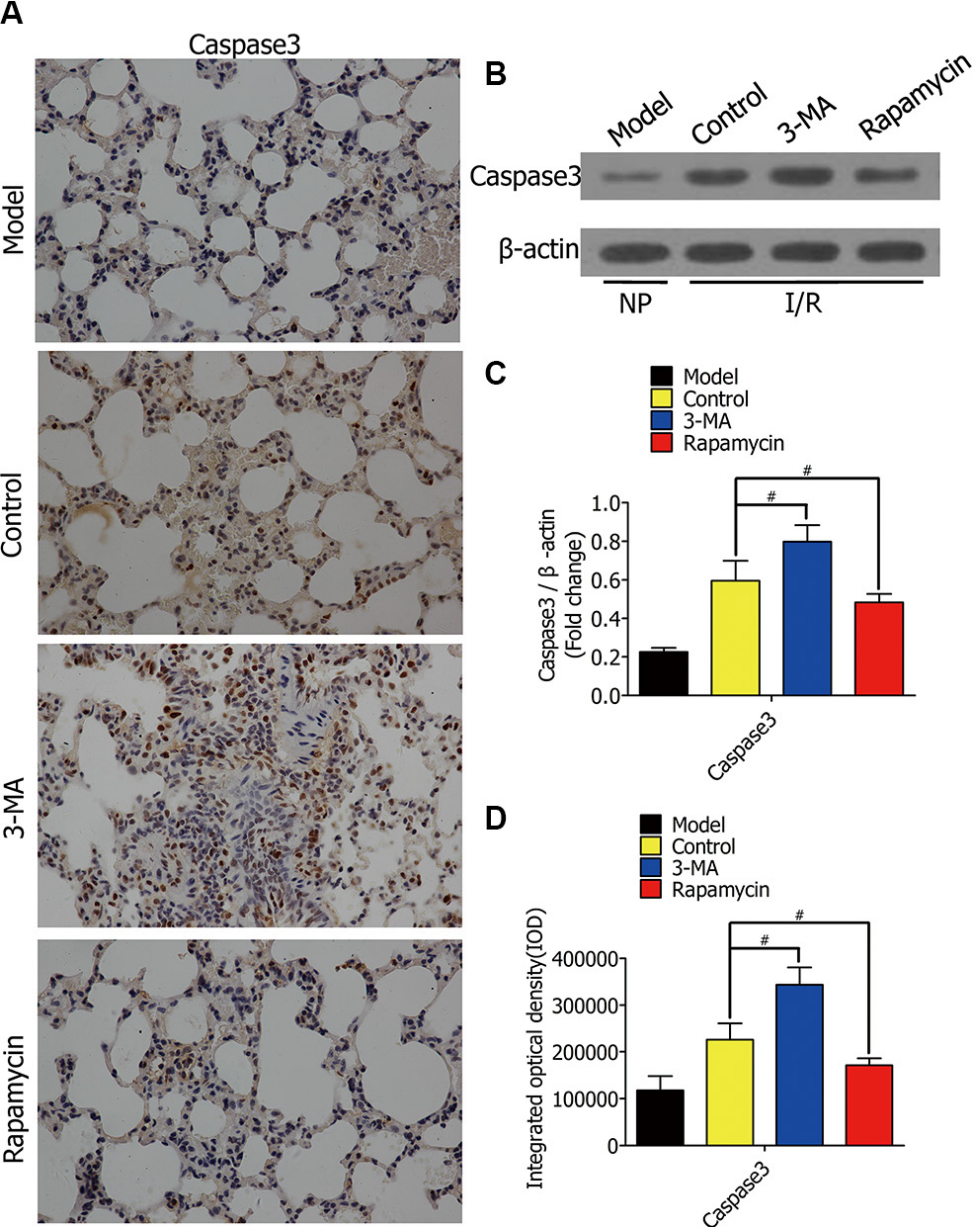
(**A**) Representative immunohistochemical staining images of normal rat lung section (model group), lung section lavaged with DMSO (control group), lung section lavaged with 3-MA (3-MA group), and lung section lavaged with rapamycin (rapamycin group), with an antibody against caspase-3. Bars represent 30 μm. (**B**) Western blots showing caspase-3 protein levels in lung tissues from Lewis rats after the rats were lavaged with DMSO, 3-MA, and rapamycin in lung ischemia for 1h and reperfusion for 2 h, respectively. The model group was not treated with ischemia. (**C**) The protein levels of caspase-3 in Lewis rats were quantified and analyzed in the indicated groups. (**D**) Immunohistochemistry analysis of the protein expressions of caspase-3 in rat lungs in the indicated groups. (^#^*p* < 0.05 compared to control).

To observe the effect of autophagy on alveolar macrophage apoptosis in rat lung I/R, we lavaged rat lungs with autophagy promotor (rapamycin) or inhibitor (3-MA)during ischemia for 1 h, followed by reperfusion for 2h.Immunohistochemical staining of the lung sections was used for evaluating caspase-3expression (Figure 7A). 3-MA enhanced caspase-3 expression in rat lung I/R injury, while rapamycin reduced it (Figure 7D). Western blotting showed the protein levels of caspase-3 in lung tissues from Lewis rats after I/R (Figure 7B). The model group was not treated with ischemia. The protein levels of caspase-3in the 3-MA group were higher than the control group, but lower in the rapamycin group (Figure 7C).

### Enhanced autophagy decreased alveolar macrophage apoptosis in rat lung I/R

**Figure 8:**
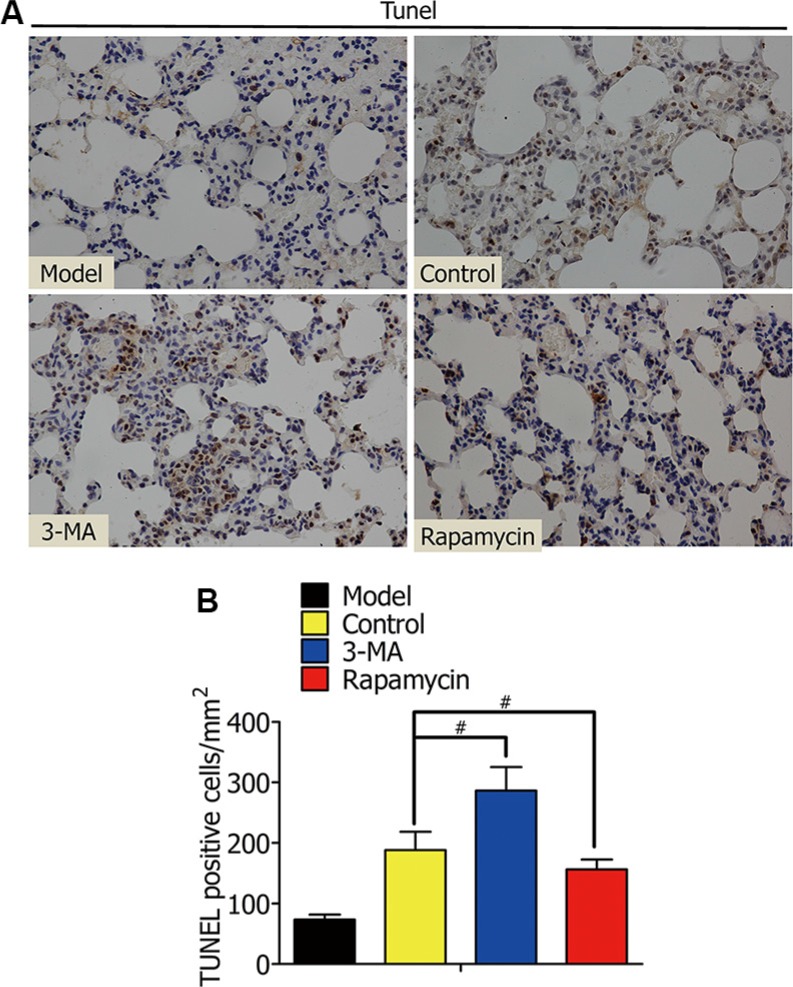
(**A**) TUNEL staining of rat lung tissue showed the apoptotic death of nuclear cells of alveolar macrophages in the model, control, 3-MA, and rapamycin groups. (**B**) Percentage of TUNEL (+) cells (apoptosis cells) in TUNEL staining. (^#^*p* < 0.05 compared to control).

To further illuminate whether the enhanced autophagy is a way of protecting alveolar macrophages in oxygen deficiency, we lavaged rat lungs with DMSO, 3-MA, or rapamycin during lung ischemia for 1 h, followed by reperfusion for 2 h. The model group was not treated with ischemia. After that, TUNEL staining of lung tissues from Lewis rats was used to assay alveolar macrophage apoptosis (Figure 8A). The percentage of TUNEL (+) cells (apoptosis cells) in the 3-MA group was higher than the control group, but lower in the rapamycin group (Figure 8B).

### Enhanced autophagy stimulated ERS in rat lung I/R

**Figure 9:**
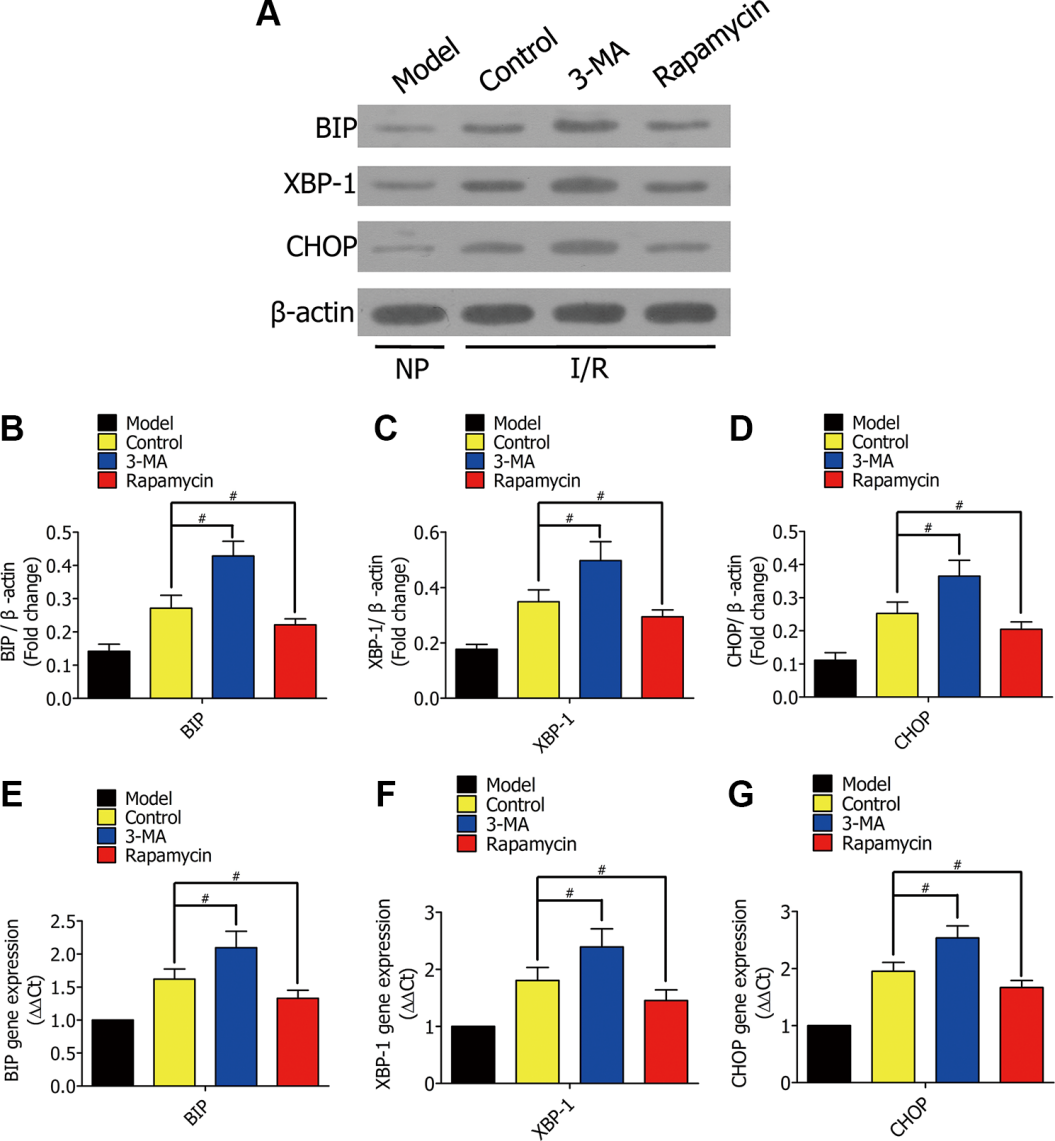
(**A**) Western blots showing the protein levels of BIP, XBP-1, and CHOP in lung tissues from Lewis rats in the model group (normal rat lung without ischemia), control group (lavaged with DMSO during ischemia), 3-MA group (lavaged with 3-MA during ischemia), and rapamycin group (lavaged with rapamycin during ischemia). (**B**–**D**) The protein of BIP, XBP-1, and CHOP in Lewis rats were quantified and analyzed in the indicated groups. (**E**–**G**) Real-time PCR showing the expression of BIP, XBP-1, and CHOP in the indicated groups. Results were presented as (2–ΔΔCt) ± SD. (^#^*p* < 0.05 compared to control).

To further verify the mechanism of enhanced autophagy decreasing alveolar macrophage apoptosis *in vivo*, we measured ERS-related genes in rat lung I/R. We lavaged rat lung with DMSO, 3-MA, or rapamycin during lung ischemia for 1h, followed by reperfusion for 2 h. Western blotting results showed the protein levels of BIP, XBP-1, and CHOP in lung tissues from Lewis rats (Figure 9A), which were quantified and analyzed in the indicated groups (Figure 9B, 9C, 9D).Real-time PCR further showed the genes expression of BIP, XBP-1, and CHOP (9E, 9F, 9G). The ERS-related gene expression was higher in the 3-MA group when compared to the control group, but lower in the rapamycin group.

### Enhanced autophagy attenuated oxidative stress in rat lung I/R injury

**Figure 10:**
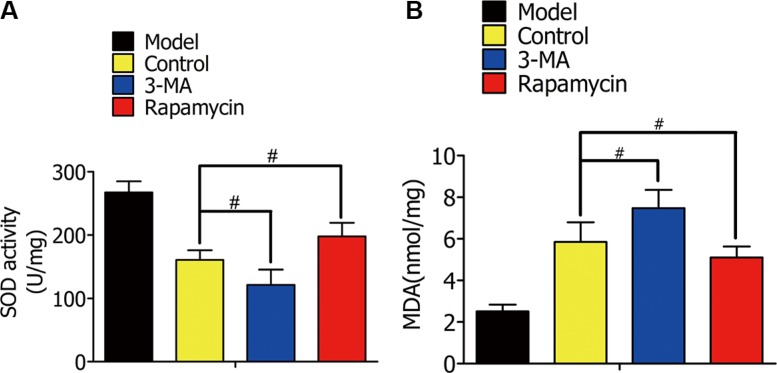
(**A**) Superoxide dismutase (SOD) activity in alveolar macrophages in the model group (normal rat lung without ischemia), control group (lavaged with DMSO during ischemia), 3-MA group (lavaged with 3-MA during ischemia), and rapamycin group (lavaged with rapamycin during ischemia). (**B**) Malondialdehyde (MDA) content in alveolar macrophages in the indicated groups. (^#^*p* < 0.05 compared to control).

In rat lung I/R, we detected MDA content and SOD activity to estimate oxidative stress levels. Rat lungs were lavaged with DMSO, 3-MA, or rapamycin during lung ischemia for 1 h, followed by reperfusion for 2 h. SOD activity in the 3-MA group was lower than the control group, and higher in the rapamycin group (Figure 10A). Contrary to the SOD levels, MDA decreased with increased autophagy (Figure 10B).

## DISCUSSION

Based on various *in vivo* and *in vitro* tissue ischemia and cell hypoxia models, we clearly showed that exogenously enhancing autophagy decreases alveolar macrophage apoptosis by reducing endoplasmic reticulum stress in the early stages of hypoxia.

Hypoxia-reoxygenation (H/R) injury is associated with multiple respiratory diseases. We observed an increase in autophagy levels, ERS, and alveolar macrophage apoptosis with prolonged H/R. When permeated with autophagy promoter (rampamycin), alveolar macrophages showed low levels of ERS, oxidative stress, and apoptosis rate in the early stages of H/R or I/R. When permeated with autophagy inhibitor (3-MA), alveolar macrophages showed high levels of ERS, oxidative stress, and apoptosis rate in the early stages of H/R or I/R.

LC3 is one of most important autophagosome membrane markers, and regarded as a key reflector of cellular autophagy activity [31, 32]. We successfully established a stable RFP-LC3/NR8383 cell line by constructing and transfectingRFP-LC3 plasmid into NR8383 cells. HDAC6 is an essential autophagy effector, reported to participate in regulation of autophagy-related cilia dysfunction during chronic obstructive pulmonary disease [33, 34]. Beclin1 was described as an essential autophagy effector and haplo-insufficient tumor suppressor [35]. To verify autophagy could be regulated by 3-MA and rapamycin, we measured the expression of autophagy-related genes LC3, Beclin1, and HDAC6. Our results reveal that the protein levels of those genes were inhibited by 3-MA, and promoted by rapamycin.

As one of many apoptosis-related genes, caspase-3 levels were elevated in the 3-MA group, and suppressed in the rapamycin group. We used MTT and TUNEL assays to further evaluate alveolar macrophage cell viability and apoptosis in early stages of H/R and rat lung I/R. The results confirmed that enhanced autophagy decreased alveolar macrophage apoptosis, and increased cell viability in an oxygen-deficient environment, both *in vitro* and *in vivo*.

ERS is the major cause of cell death during hypoxia. We showed the protein levels of BIP, XBP-1, and CHOP decreased in response to rapamycin, and increased in response to 3-MA. Quantification of SOD and MDA provide an accurate assessment of oxidative stress, both *in vitro* and *in vivo*. 3-MA decreased SOD activity, while rapamycin increased it. Therefore, exogenously enhancing autophagy decreases alveolar macrophage apoptosis by reducing endoplasmic reticulum stress and oxidative stress in the early stages of hypoxia, both *in vitro* and *in vivo*.

Using autophagy inhibitor (3-methyladenine, 3-MA) and autophagy promoter (rapamycin) to control autophagy levels, we demonstrate that exogenously enhancing autophagy decreases alveolar macrophage apoptosis by attenuating endoplasmic reticulum stress and oxidative stress, which could be a new protective method in lung ischemia-reperfusion injury.

## MATERIALS AND METHODS

### Cell culture

For *in vitro* studies, the alveolar macrophage NR8383 (ATCC, CRL-2192) cell line was chosen as a cell model. The cells were maintained in F-12K medium at 37°C in a humidified 5%CO2 atmosphere. Additionally, the medium contained 10% heat-in activated fetal calf serum. When the cells reached 80% confluence, they were digested with 0.25% trypsin.

### Constructing stable RFP-LC3/NR8383 cell line

TheRFP-LC3plasmid was transfected into NR8383 cells by applying Lipofectamine 2000 reagent. Twenty-four hours later, the cells were transferred to culture in F-12K medium containing 600 μg/ml of G418. After 2 weeks of expansion, the NR8383cellswere observed under a fluorescence microscope (Olympus Japan), and the strong red fluorescent colonies were selected as stable RFP-LC3/NR8383 cell and cultured in the medium containing 100 μg/ml G418 and 10% FBS for further experiments in the study.

### Animal models and procedures

All the animal experimental protocols were approved by the Animal Care and Use Committee of Renmin Hospital of Wuhan University, and were conducted in accordance with the National Institutes of Health (NIH) Guide for the Care and Use of Laboratory Animals.

Sprague Dawley (SD) male rats (8 weeks old, 250 to 300 g) were fed with a standard diet and maintained in a controlled environment of the animal center. Rats were anesthetized by an intraperitoneal injection of 10% chloral hydrate (300 mg/kg body weight), and placed in a supine position. The animals were then intubated for artificial ventilation with oxygen using a small animal breathing machine (tidal volume 5 ml, frequency 70 per min) and electrocardiograph monitor. A thoracotomy was performed at the anterior lateral side of the left fourth intercostal. The muscular layer and pleura were gently dissected to expose the heart and lung. The hilum of the left lung was dissociated, and the artery clamp was used to pass through the hilum of the lung from the upper right to the lower left. The whole clamped left hilum was clearly exposed by slightly stirring up the clamp.

Twenty SD rats were randomly divided into four groups (5 rats/group) as follows: (1) model group: no blocking of hilum in left lung; (2) control group: blocking of hilum in left lung for 1 h with DMSO lavage, and then reperfusion for 2 h; (3)3-MA group: blocking of hilum in left lung for 1 h with 100 ml/kg 3-MA (5 μmol/L) solution lavage, and then reperfusion for 2 h; (4) rapamycin group: blocking of hilum in left lung for 1 h with 100 ml/kg rapamycin (250 nmol/L) solution lavage, and then reperfusion for 2 h. The rats in all four groups were sacrificed after the experiments, but the left lung tissue was dissected for further analysis.

### Immunofluorescence analysis

The surface area of RFP-LC3/NR8383 cells was assessed by immunofluorescent staining. The cells were fixed with 4%formaldehyde, permeabilized with 0.1%Triton X-100/BS for 45 min, stained with β-actin (1:100 dilution), followed by a fluorescent secondary antibody. Images were captured using a fluorescence microscope(Olympus, Japan), and the surface areas were measured using *Image-Pro Plus 6.0* software.

### Immunohistochemical analysis

For immunohistochemistry *in vitro*, RFP-LC3/NR8383 cells growing on glass coverslips were fixed for15min with 4%paraformaldehyde. Cells incubated with a 0.5%Triton X-100/PBS solution for 30minand washed three times with PBS. They were then blocked with 3% hydrogen peroxide for 15 min and incubated overnight at 4°C with primary antibodies. Binding was visualized with the appropriate peroxidase-conjugated secondary antibodies (AR1022, ZSGB-BIO) for 20 to 30 min at 37°C.

For immunohistochemistry *in vivo*, paraffin-embedded lungs were cut transversely into 5-μm sections. After a 5-min high-pressure antigen retrieval process in 0.1 mol/L citrate buffer PH 6.0, the lung sections were blocked with 3% hydrogen peroxide for 15 min and incubated overnight at 4°C with the primary antibodies. Binding was visualized with the appropriate peroxidase-conjugated secondary antibodies (AR1022, ZSGB-BIO) for 20 to 30 min at 37°C.

### Western blotting analysis

Total protein was extracted from rat lung tissues and RFP-LC3/NR8383 cells in lysis buffer. Protein concentrations were determined using a Pierce BCA Protein Assay kit. Fifty micrograms of protein was subjected to SDS–polyacrylamide gel electrophoresis (12% PAGE; Amresco), transferred to a polyvinylidene fluoride membrane (Millipore), and incubated with the corresponding primary antibodies overnight at 4°C. After incubation with peroxidase-conjugated secondary antibodies (BA1051, 1:50,000 dilution), the bands were visualized using Bio-Rad ChemiDoc^™^ XRS+(Bio-Rad). Protein levels were normalized to corresponding β-actin levels.

### MTT assay

The viability of cells was measured using an MTT assay [36]. The percent cell viability inhibition was calculated as: Cell viability = [OD(treated)–OD(control)] /OD(control)× 100.

### TUNEL analysis

For *in vitro* analysis, RFP-LC3/NR8383 cells grown on glass coverslips were fixed with 4% paraformaldehyde (pH7.4) for 15 min and washed twice with PBS at room temperature. After incubated with0.2% Triton X-100/PBS solution for 5 min and was hed twice with PBS, DNA fragmentation was determined by terminal deoxynucleotidyl transferase-mediated dUTP nick end labeling (TUNEL) as described by the manufacturer. Hematoxylin Staining Solution (Sigma, H9627) was used as nuclear counterstain for the fluorescent quantification of DNA content. A total of 5 high power fields (×400 magnification) in every group were randomly selected in a blinded manner. In each field, cells with apparent TUNEL nuclear staining (brown) represented TUNEL-positive cells. RFP-LC3/NR8383 cell apoptosis was expressed as apoptotic index (AI) calculated as follows: AI = TUNEL-positive cells/mm^2^.

For *in vivo* analysis, identification of apoptotic nuclei was carried out with the TUNEL method as well. The rat lung tissues were cut transversely into 5-μm sections and fixed with 4%paraformaldehyde (pH7.4) for 15 min. After two PBS washes at room temperature, sections were permeabilized using a 0.2% solution of Triton X-100/PBS. DNA fragmentation was determined by TUNEL. Hematoxylin Staining Solution (Sigma, H9627) was used as nuclear counterstain for the fluorescent quantification of DNA content. A total of 5 high power fields (×400 magnification) in every group were randomly selected in a blinded manner. In each field, cells with apparent TUNEL nuclear staining (brown) represented TUNEL-positive cells. RFP-LC3/NR8383 cells apoptosis was expressed as apoptotic index (AI) calculated as follows: AI = TUNEL-positive cells/mm^2^.

### Measurement of superoxide dismutase (SOD) activity

For *in vitro* analysis, RFP-LC3/NR8383 cell lysis buffer was mixed with 10% SOD buffer, Triton X-100 (0.4%v/v), and 200 μM phenylmethanesulfonyl fluoride, then centrifuged at 10,000 × g for 5 min at 4°C to obtain supernatant. For *in vivo* analysis, each tissue was homogenized in PBS (pH7.4), then centrifuged at 400 × g for 5min at 4°C. The pellets were resuspended in 5 volumes of ice-cold 1X cell extraction buffer [10% SOD buffer, Triton X-100 (0.4% v/v), and 200 μM phenylmethanesulfonyl fluoride], then centrifuged at 10,000 × g for 5 min at 4°C to obtain supernatant.

The SOD activities in the supernatants from both methods were measured using assay kits per the manufacturer's instructions. The amount of SOD in the extract was determined by specific enzyme activity (U/mg) utilizing a commercial SOD as standard.

### Measurement of malondialdehyde (MDA) contents

For *in vitro* analysis, RFP-LC3/NR8383 cells were mixed with lysis buffer; for *in vivo* analysis tissue was homogenized in PBS (pH7.4). Both were centrifuged at 10,000 × g for 5min at 4°C. Each supernatant was incubated with 3 mLH_3_PO_4_ solution (1%w/v) and 1 mL thiobarbituric acid solution (0.67% w/v) for 40 min in a 95°C water bath. After cooling, the reactants were centrifuged at 1,000 × g for 5min. The supernatants were measured at 532 nm using a spectrophotometer. MDA contents were presented as nmol/mg of protein [37].

### RNA isolation and Real-time PCR

Total mRNA was extracted using Trizol reagent (15596-018, Invitrogen) per the manufacturer's instructions. mRNA was converted to cDNA using oligo primers with a Transcriptor First Strand cDNA Synthesis Kit (#K1622, Fermentas). Quantitative real-time PCR amplification of the indicated genes was performed using SYBR Green/FluoresceinqPCR 2X Master Mix (#K0242, Fermentas). Target gene expression was normalized to β-actin gene expression. Reverse transcription thermal conditions were as follows: 60 min at 42°C, followed by 5 min at 95°C. The following primers were used to analyze gene expression: β-actin Fwd 5′-CAC GAT GGA GGG GCC GGA CTC ATC-3′ and Rwd 5′-TAA AGA CCT CTA TGC CAA CAC AGT-3′; BIP Fwd 5′- AGCCCACCGTAACAATCAAG-3′ and Rwd 5′- CCTGTCCCTTTGTCTTCAGC-3′; XBP-1 Fwd 5′- CCC ATG GAT TCT GAC GCT GT-3′ and Rwd 5′- ATGAGGTCCCCACTGACAGA-3′; CHOP Fwd 5′- CAGCGCATGAAGGAGAAGGA-3′ and Rwd 5′- ACAGGAGGTGATGCCAACAG-3′.

### Statistical analysis

Data are presented as the mean ± s.d. from at least three independent experiments. Student's two-tailed *t*-test was used to compare the means of two-group samples. Two-way analysis of variance (ANOVA) was applied for comparison of multiple groups with different H/R times. A one-way analysis of variance (ANOVA) was applied to determine the effect of 3-MA or rapamycin on studied rats pretreated with lung I/R, followed by the least significant difference (equal variances assumed) or Tamhane's T2 (equal variances not assumed) tests. All statistical analyses were performed with *Graph Pad Prism5* software. *P* values less than 0.05 were considered significant. No statistical method was used to predetermine sample size. Randomization and blinding strategies were used whenever possible.
